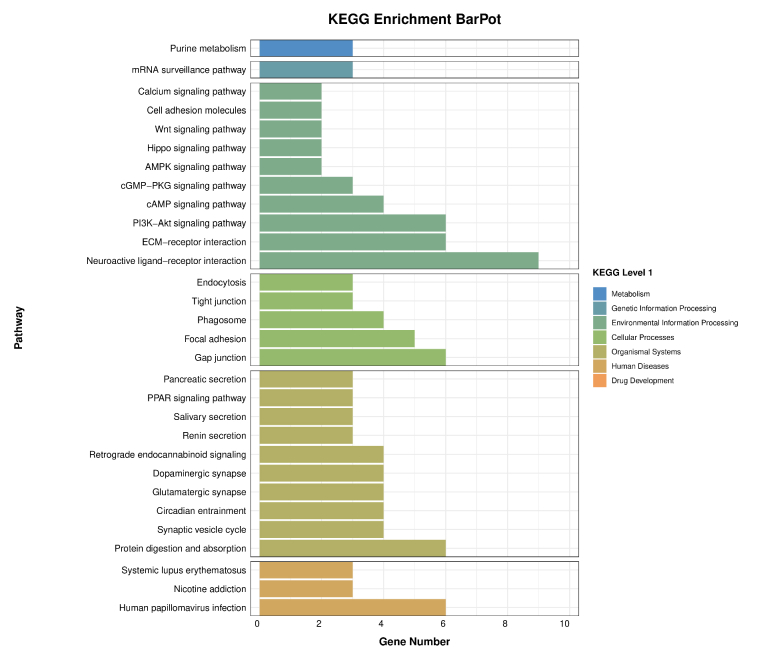# Loss of Hdac4 in osteoprogenitors impairs postnatal trabecular and cortical bone formation, resulting in a dwarfism and osteopenia phenotype in mice

**DOI:** 10.1016/j.jbc.2024.107941

**Published:** 2024-10-29

**Authors:** YunFei Wang, Raorao Zhou, Zhengquan Dong, Wenting Wang, Li Guo, Jian Sun, Xueqin Rong, Pengcui Li

**Affiliations:** 1 Department of Surgery, Shanxi Bethune Hospital, Shanxi Academy of Medical Science, Tongji Shanxi Hospital, Third Hospital of Shanxi Medical University, Taiyuan, China; 2 Department of Orthopedics, The Second Hospital of Shanxi Medical University, Shanxi Key Laboratory of Bone and Soft Tissue Injury Repair, Taiyuan, China; 3 Department of Biochemistry and Molecular Biology, Shanxi Key Laboratory of Birth Defect and Cell Regeneration, Shanxi Medical University, Taiyuan, China; 4 Department of Pain Spinal Minimally Invasive Centre, Sanya Central Hospital, Sanya, Hainan, China

**Keywords:** hdac4, osteoprogenitors, postnatal osteogenesis, dwarfism, osteopenia

## Abstract

HDAC4 is a class II histone deacetylation protein with a well-characterized role in chondrocyte differentiation and skeletal development, and dysregulated expression or haploinsufficiency of Hdac4 leads to skeletal formation and malformation disorders. The early lethality of Hdac4 ablation mice hindered further investigation of its role in postnatal bone growth and development. Therefore, this study aims to investigate the significant role of Hdac4 in postnatal endochondral bone development using two mouse models with conditional deletion of Hdac4 in Sp7-expressing osteoprogenitors or chondrocytes and monitored postnatal bone development. The phenotype of *Acan-Cre^ERT2^; Hdac4*^*fl/fl*^ mice largely resembled that of conventional *Hdac4*^*−/−*^ mice. But phenotypic characterizations of mice with Hdac4 inactivation in Sp7-expressing osteoprogenitors (*Sp7-Cre; Hdac4*^*fl/fl*^) showed dwarfism with body and limb shortening and remarkable skeletal defects. Microcomputed tomography analysis of tibias further demonstrated that loss of Hdac4 expression impaired bone formation and microarchitecture, mainly characterized by dysplasia of trabecular and cortical bone in young mice. Our *in vivo* and *in vitro* data support a crucial role for Hdac4 in regulating osteoblast proliferation and differentiation, bone matrix protein production, angiogenesis, and ultimately trabecular and cortical bone formation. Moreover, RNA-seq analysis implicated Hdac4 in the regulation of key genes and pathways necessary to affect the accumulation of extracellular matrix, biological processes related to signal transduction, and skeletal growth. Collectively, our data show that postnatal expression of Hdac4 in Sp7-expressing osteoprogenitors provides essential regulatory oversight of endochondral bone formation, bone morphology, and homeostasis.

Most mammalian skeletons, including the ribs, sternum, axial bones, and limb bones, undergo endochondral ossification. This process involves matrix-secreting chondrocyte proliferation, cartilage template formation, and coordination of subsequent bone formation (1, 2). During prenatal and postnatal bone development, perichondral/periosteal ossification occurs, along with the migration of blood vessels and osteoblasts. These elements work together to deposit bone to replace the growth plate (3). The transition from cartilage to bone and further development involves a succession of synchronous events and requires the close coupling of chondrocyte differentiation, osteoblast deposition, and angiogenesis. Osteogenesis is a critical process reliant on osteoblast activity and necessitates the sequential and tightly regulated expression of osteogenic-related genes (4). Dysregulation of essential signaling pathways and transcription factors in endochondral ossification can result in various skeletal dysplasias and patterning defects, including conditions such as kyphomelic dysplasia, rib cage deformity, brachydactyly, osteoporosis, and osteosclerosis.

Histone deacetylases (HDACs) are a group of enzymes with deacetylation activity, categorized into four classes (I, II, III, and IV), playing a key role in gene expression regulation through histone modifications and chromatin structural remodeling in various biological processes. These enzymes also possess a lengthy N-terminal extension that connects extracellular signals with the cell genome by interacting with transcription factors, such as Mef2c, Atf4, and Runx2 (5, 6). Among the class II family, HDAC4 is a significant member, predominantly expressed in osteoblast lineage cells and prehypertrophic chondrocytes (7, 8). Significantly, Vega *et al.* reported that Hdac4^−/−^ mice exhibit premature and misplaced endochondral ossification, accompanied by accelerated hypertrophic chondrocytes, resulting in a lethal dwarfism phenotype (8). Their study demonstrated that Hdac4 inhibits Runx2 expression and regulates chondrocyte hypertrophy during bone development. Human studies align with these findings, as HDAC4 haploinsufficiency is associated with a skeletal phenotype characterized by brachydactyly type E, developmental delays, and mental retardation (9). Studies have shown that HDAC4 expressed by chondrocytes can transmit signals to the proliferating region of growth plates, influencing chondrocyte proliferation (10). This signaling also contributes to angiogenesis and premature ossification (11). In osteoblasts, Hdac4 expression does not inhibit Runx2 but rather integrates with PTH and sympathetic signaling, affecting various physiological processes in mice, such as metabolism, spatial learning and memory, and insulin secretion (12, 13, 14). Mice with mature osteoblasts and an osteocyte-specific deletion of Hdac4 are essentially indistinguishable from normal mice and exhibit no skeletal phenotypes (15). Our previous laboratory studies have uncovered a relationship between Hdac4 and cartilage degeneration and chondrocyte hypertrophy, contributing to the expression of osteoarthritis-related genes (16, 17). The thorough understanding of the role of Hdac4 in bone biology could make a significant contribution to potential avenues for therapeutic interventions in bone-related disorders. However, owing to the early lethality observed in global Hdac4 deletions or chondrocyte KO mutants, the role of Hdac4 expressed by osteoblast lineage cells in postnatal bone formation, specifically its effect on osteoblast differentiation and osteogenesis, remains unclear. Additionally, distinguishing the early effects of Hdac4 on osteogenesis and skeletal remodeling from its concurrent effects on chondrocytes is challenging.

Therefore, this study aims to investigate the significant role of Hdac4 in postnatal endochondral bone development using two conditional KO mouse models (*Sp7-Cre;Hdac4*^*fl/fl*^ and *Acan-Cre*^*ERT2*^*;Hdac4*^*fl/fl*^). Our findings indicate that the deletion of Hdac4 in osteoblast lineage cells does not affect prenatal bone development. However, it significantly contributes to postnatal bone formation, leading to a dwarfism phenotype in adult mutant mice characterized by pronounced trabecular and cortical bone dysplasia.

## Results

### Generation and gross phenotypes of *Sp7-Cre; Hdac4*^*fl/fl*^ mice

**Figure 1:**
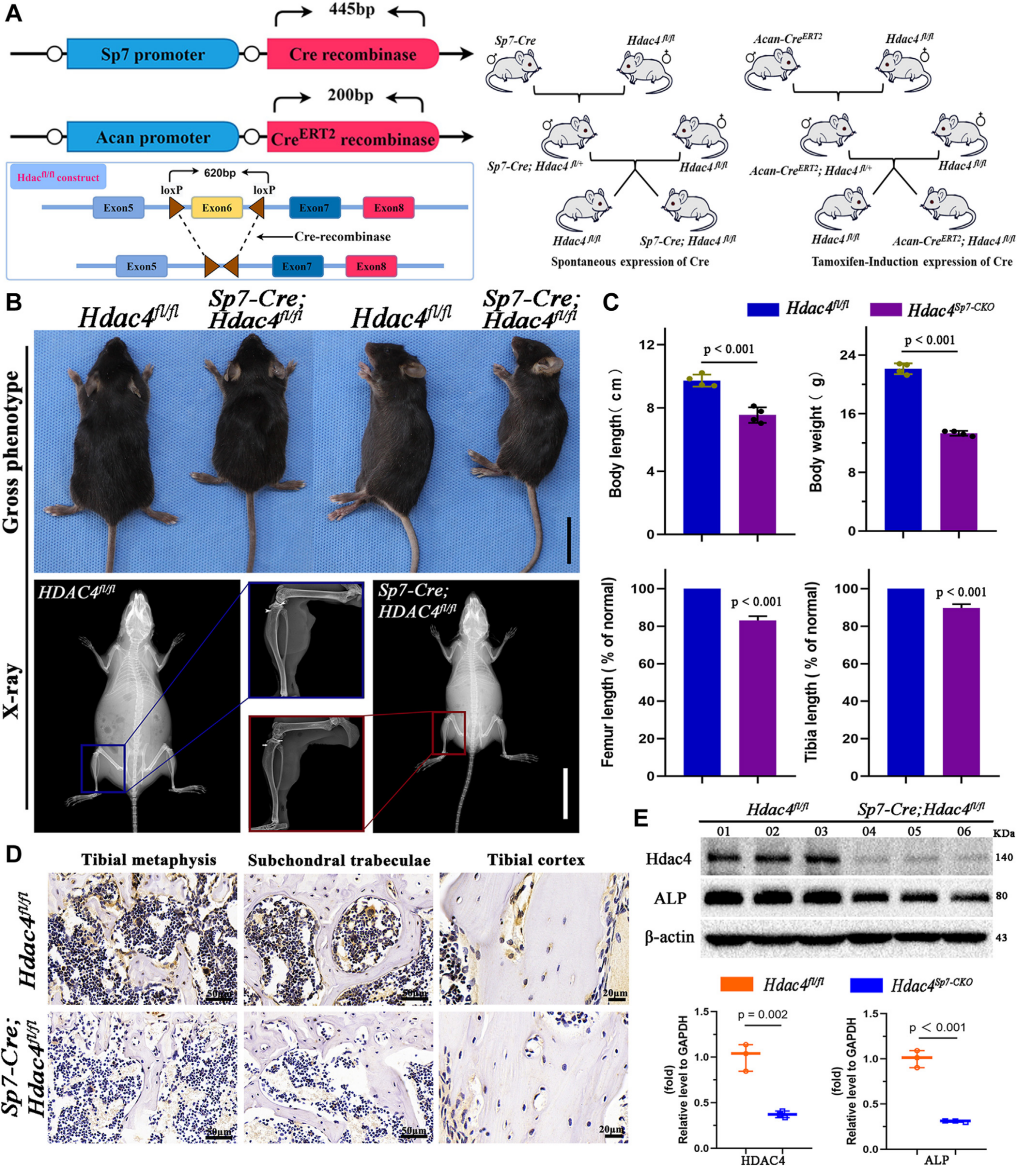
***Sp7-Cre; Hdac4***^***fl/fl***^**transgenic mice were successfully generated and developed dwarfism with skeletal dysplasia.***A*, schematic representation and breeding strategies of the *Sp7-Cre; Hdac4*^*fl/fl*^ transgenic mice and *Acan-Cre*^*ERT2*^*; Hdac4*^*fl/fl*^ transgenic mice. *B*, the Gross phenotype and representative X-ray images of 2-month-old male control and *Sp7-Cre; Hdac4*^*fl/fl*^. Mutants exhibit a dwarf body and short limbs. *Purple* or *blue boxes* indicate magnified areas, showing short hindlimbs, and osteopenia (*arrow*). *Black bars* represent 2 cm, *white bars* represent 2 cm. *C*, measurement of body weight and length of 2-month-old mice (n = 4). Measurement of femur length (% of normal femur length) (n = 3), the length of tibia (% of normal tibia length) (n = 3). Between-group comparisons utilized Student’s t-tests. The *p*-values for statistical comparisons between groups are shown in the Figure. *D*, immunohistochemistry staining of Hdac4 on paraffin sections of 2-month-old *Hdac4*^*fl/fl*^ or *Sp7-Cre; Hdac4*^*fl/fl*^ tibias. The results showed that Hdac4 protein was weakly expressed both in the tibial metaphysis, subchondral trabeculae, and tibial cortex. Bar represents 50 μm or 20 μm. *E*, Western blots and RT-qPCR show the protein and mRNA expression levels of Hdac4 and ALP in tibias derived from 2-month-old *Hdac4*^*fl/fl*^ or *Sp7-Cre; Hdac4*^*fl/fl*^, respectively. β-actin served as an internal control. Data are the mean ± SD, n = 3. Table S1 presents the primer sequences used for the RT-qPCR.

**Supplemental Fig. S1:**
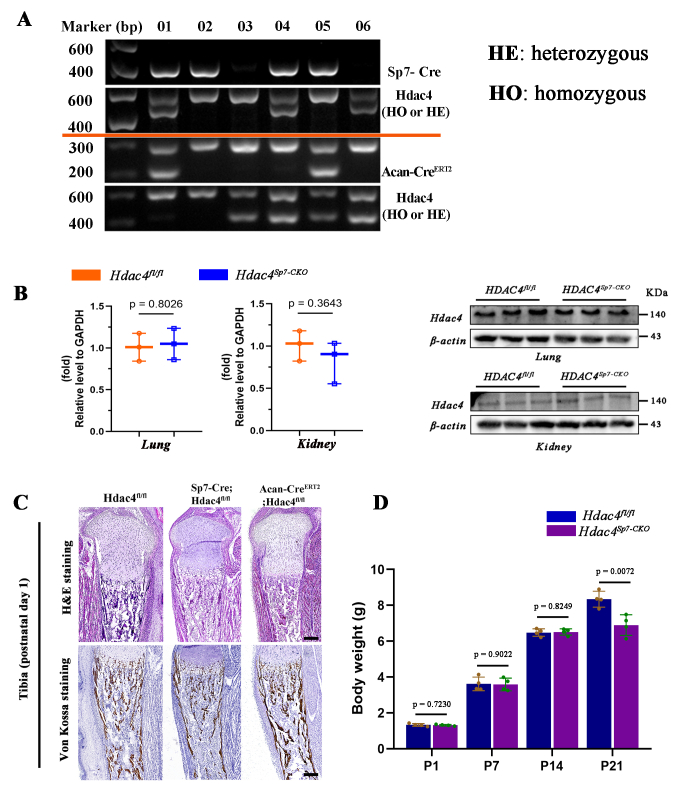


Sp7, also known as osterix, is commonly used as an indicator of osteoblastic lineage cells expressed from the osteoprogenitor stage onwards. As reported previously, Sp7-Cre efficiently facilitates Cre recombinase activity in osteoblast progenitors, targeting the entire osteoblastic lineage (18, 19). To explore the role of Hdac4 in postnatal bone development, we crossed *Sp7-Cre* transgenic mice with a floxed Hdac4 animal background (*Hdac4*^*fl/fl*^) to generate *Sp7-Cre;Hdac4*^*fl/fl*^ mice. Figures 1*A* and S1*A* present the breeding strategies and genotypic identification of the mice. *Sp7-Cre;Hdac4*^*fl/fl*^ mice adhered to the Mendelian ratios and displayed a generally normal phenotype (Fig. S1*C*). Both male and female *Sp7-Cre;Hdac4*^*fl/fl*^ mice at 2 months exhibited smaller body sizes than their control littermates. Specifically, the body weight and length measured from the nose to tail of *Sp7-Cre;Hdac4*^*fl/fl*^ mice were significantly lower and shorter, respectively than those of the control mice. In addition, X-ray measurements of tibia length further revealed an apparent decrease in bone length and bone mineral density in *Sp7-Cre;Hdac4*^*fl/fl*^ mice compared to *Hdac4*^*fl/fl*^ mice (Fig. 1, *B* and *C*). Expression analysis of alkaline phosphatase (ALP) (the most recognised marker of osteoblast activity) revealed reduced osteoblast activity in mutant mice. Expression analysis, utilizing RNA and proteins isolated from the hind limbs of *Hdac4*^*fl/fl*^ and *Sp7-Cre;Hdac4*^*fl/fl*^ mice at 2 months, confirmed a significant deletion of Hdac4 in the mutant mice (Fig. 1, *D* and *E*). In contrast, the mRNA levels of Hdac4 in the kidneys and lungs of control and mutant mice were comparable. Western blotting also confirmed that Hdac4 in the kidneys and lungs of mutant and control mice were comparable in protein levels (Fig. S1*B*).

### Sp7-Cre–mediated deletion of Hdac4 leads to reduced osteogenesis and bone mass with changes in micro-structure

**Figure 2:**
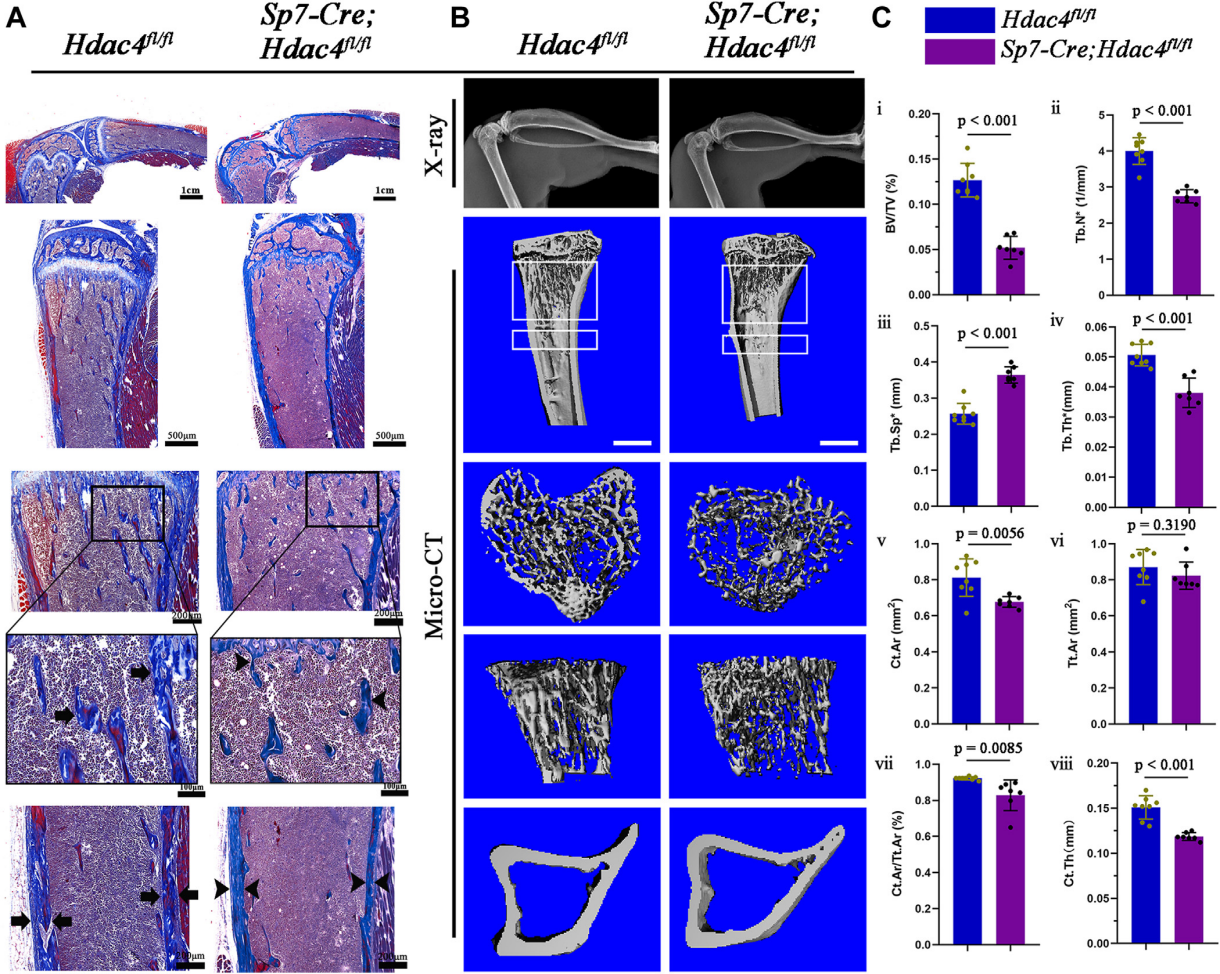
**Loss of Hdac4 in Sp7-expressing osteoprogenitors resulted in reduced cortical and cancellous bone formation in the 2 months tibias.***A*, representative images of Masson's trichrome staining showed that sparse trabecular bone and thin cortical bone were observed in *Sp7-Cre; Hdac4*^*fl/fl*^ mice at 2 months. Scale bars are shown in the correspondence diagram. *Black arrows* or *arrowheads* indicate trabeculae and cortex of the long bone in two groups of mice, respectively. *B*, representative X-ray images showing whole tibias (*upper panel*); representative micro-CT images of tibias from 2-month-old mice (*lower panel*) show the proximal tibia (*top*; scale bars represent 1 mm), trabecular bone of the tibia metaphysis (*middle*), and cortical (*bottom*). *C*, indicators for evaluating the spatial morphologic structure of bone trabeculae (i–iv): bone volume as a percentage of total volume (BV/TV, %), trabecular thickness (Tb. Th, mm), number of trabeculae (Tb. N, 1/mm), and trabecular separation (Tb. Sp, 1/mm). Cortical bone was analyzed as follows (v–viii): total cortical bone area (Tt. Ar, mm^2^), cortical bone area (Ct. Ar, mm^2^), cortical bone thickness (Ct. Th, mm), and cortical bone area as a percentage of total area (Ct. Ar/Tt. Ar, %). *Hdac4*^*fl/fl*^ mice: n = 8; *Sp7-Cre; Hdac4*^*fl/fl*^ mice: n = 7. Data are the mean ± SD, the *p*-values for statistical comparisons between groups are shown in the Figure.

Given the dwarfism observed in the mutant mice and the significantly shorter limb length than that of WT mice at 2 months of age, we examined the histomorphological characteristics of the tibial bone using Masson’s trichrome staining. The staining revealed that the mutant mice exhibited sparse trabecular bone in the primary ossification center and reduced thickness of the cortical bone, as indicated by the black arrow and arrowhead in Figure 2*A*. Additionally, μCT analysis was performed to determine the bone microstructure of the tibias (Fig. 2*B*). Skeletal analysis revealed that Hdac4 cKO mice also showed a phenotype with significantly lower bone mass in both cortical and trabecular bones than in *Hdac4*^*fl/fl*^ littermates (Fig. 2*C*). Trabecular bone volume *versus* total volume (BV/TV), trabecular number (Tb. N∗), and trabecular thickness (Tb. Th∗) in the tibias of mice were smaller than those in *Hdac4*^*fl/fl*^ mice and were accompanied by a significant increase in trabecular separation (Tb. Sp∗). Consistent with trabecular bone, the cortical bone area (Ct.Ar), cortical area fraction (Ct.Ar/Tt.Ar), and cortical thickness (Ct.Th) also exhibited statistically significant decreases in the cortical bone, although the trend of decrease in the total cortical area (Tt.Ar) was not significantly different. Overall, these findings suggest a decrease in cortical and trabecular bone mass owing to conditional knockout of Hdac4, specifically in osteoblastic lineage cells.

### Sp7-Cre–mediated Hdac4 deletion mildly affects postnatal bone development in preweaning mice without affecting the growth plate and secondary ossification center formation

**Figure 3:**
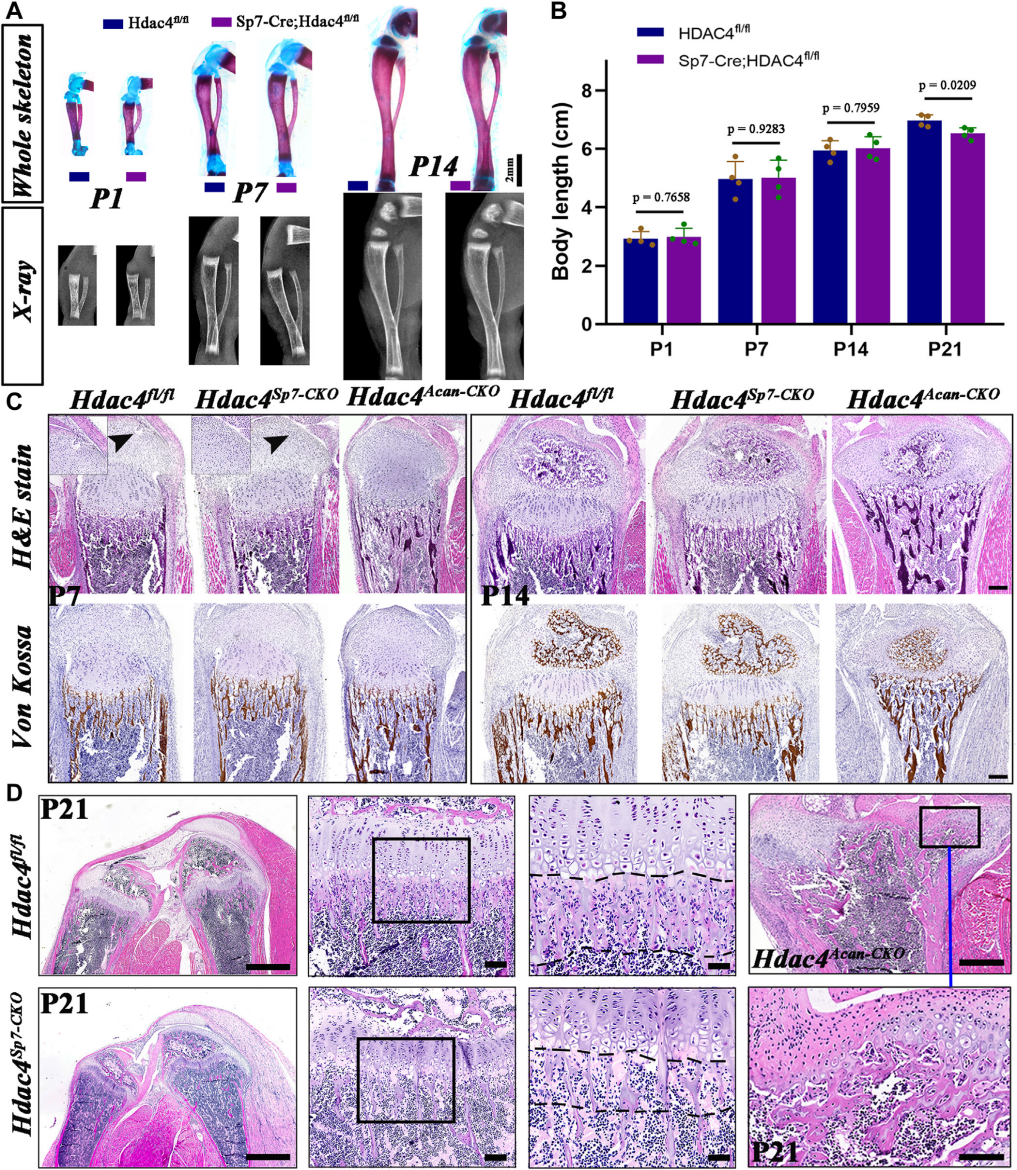
**Loss of Hdac4 in Sp7-expressing osteoprogenitors mildly affects postnatal bone development in pre-weaned mice but allows the formation of growth plates and secondary ossification centers.***A*, serial analyses of postnatal tibia phenotype using whole skeleton staining and X-ray image in *Hdac4*^*fl/fl*^ and *Sp7-Cre; Hdac4*^*fl/fl*^ mice at P1, P7, and P14. Bars represent 2 mm. *B*, measurement of body length in the mice of different ages. n = 4. Data are the mean ± SD, the *p*-values for statistical comparisons between groups are shown in the Figure. *C*, H&E stain and Von Kossa stain of longitudinal tibia sections from *Hdac4*^*fl/fl*^, *Sp7-Cre; Hdac4*^*fl/fl*^ and *Acan-Cre*^*ERT2*^*; Hdac4*^*fl/fl*^ mice at P7, P14. As shown, *Sp7-Cre;Hdac4*^*fl/fl*^ mice displayed delayed onset of cartilaginous canal at P7, weak mineral deposition in primary ossification centers of tibias at P14, but thinner growth plates and strong mineralization of primary sponges were observed in the corresponding region of the *Acan-Cre*^*ERT2*^*; Hdac4*^*fl/fl*^. *D*, H&E stain showed that a decrease in epiphyseal osteogenesis were observed in *Sp7-Cre; Hdac4*^*fl/fl*^ mice, but a complete loss of the growth plate, accelerated ossification of the epiphysis in *Acan-Cre*^*ERT2*^*; Hdac4*^*fl/fl*^ tibias at P21. Bars represent 200 μm in the *upper panels*; bar represents 50 μm in the magnified panels.

Previous studies revealed the role of Hdac4 as a central regulator of growth plate development and bone formation. To explore the independent functions of Hdac4 in osteoblast lineage cells during postnatal bone development, we crossed *Hdac4*^*fl/fl*^ mice with *Sp7-Cre* mice, where the Cre recombinase is predominantly expressed in osteoblast lineage cells, and *Acan-Cre*^*ERT2*^ mice, where the Cre recombinase is expressed in chondrocytes. At P1 (Fig. S1*C*), *Sp7-Cre; Hdac4*^*fl/fl*^, *Acan-Cre*^*ERT2*^*; Hdac4*^*fl/fl*^, and control mice exhibited no morphological changes in the tibias. Skeletal staining and X-ray imaging in the mice of different ages further revealed that the length of *Sp7-Cre; Hdac4*^*fl/fl*^ tibias were comparable to those of *Hdac4*^*fl/fl*^ mice, the normal formation of secondary ossification centers, and longitudinal growth of the tibia were virtually unaffected (Fig. 3*A*). No significant changes in the weight, size, or length of the mutant animals were observed until 14 days after birth (Figs. 3*B* and S1*D*). Although *Sp7-Cre; Hdac4*^*fl/fl*^ mice maintained a normal phenotype until postnatal day 14, von Kossa staining showed that calcium compounds, indicative of matrix mineralization markers, were slightly weaker in the POC area of *Sp7-Cre; Hdac4*^*fl/fl*^ mice at P14 (Fig. 3*C*). Nevertheless, 1 week later, at P21, *Sp7-Cre; Hdac4*^*fl/fl*^ mice exhibited shorter body lengths and lower weights than control littermates (Figs. 3*B* and S1*D*), accompanied by a reduction in the width of the growth plates and thickness of the epiphysis, indicating a decrease in epiphyseal osteogenesis. Then we generated *Acan-Cre*^*ERT2*^*; Hdac4*^*fl/fl*^ mice and examined the effects of chondrocyte-specific Hdac4 deletion on postnatal growth plate development. *Acan-Cre*^*ERT2*^*; Hdac4*^*fl/fl*^ mice were born at the expected Mendelian rate, they did not show any abnormal phenotypes. After being injected with a single dose of 20 μl of tamoxifen solution at P1, no changes in the phenotype of the mutant tibias were evident until P7 (Fig. 3*C*). Histological sections revealed an abnormal process, starting with an initially visible thinning of the *Acan-Cre*^*ERT2*^*; Hdac4*^*fl/fl*^ tibial growth plate on postnatal days 12.5 (Fig. 3*C*), ultimately leading to complete loss of the growth plate and accelerated mineralization and ossification of the epiphysis in mutant tibias at P21(Fig. 3*D*). These changes in the mutant tibia resulted in an abnormally shaped tibia with complete loss of the semicircular articular surface (Fig. 3*D*), a feature observed in universal Hdac4-KO mice in previous studies (8, 10). By comparing the phenotypes of *Sp7-Cre; Hdac4*^*fl/fl*^ and *Acan-Cre*^*ERT2*^*; Hdac4*^*fl/fl*^ mice, we suggest that the deletion of Hdac4 in chondrocytes affects the growth plate and accelerates ossification, whereas the loss of Hdac4 in Sp7-expressing osteoprogenitors predominantly impairs postnatal endochondral osteogenesis. Unfortunately, we did not acquire older *Acan-Cre*^*ERT2*^*; Hdac4*^*fl/fl*^ mice for further experiments.

### Sp7-Cre–mediated deletion of Hdac4 leads to reduced osteoblast populations and impaired metaphyseal trabecular bone formation

**Figure 4:**
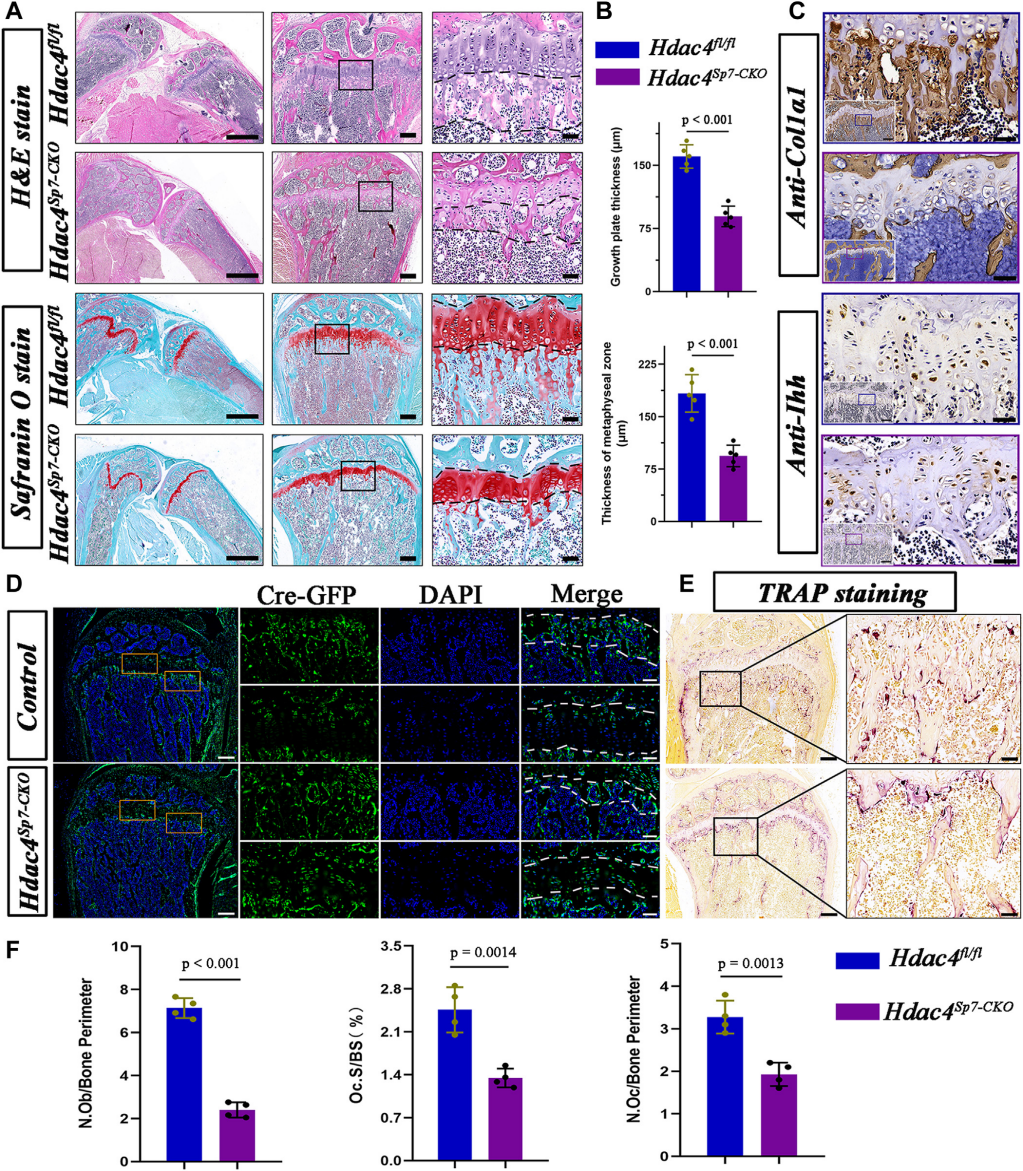
**Loss of Hdac4inSp7-expressing osteoprogenitors leads to reduced trabecular bone formation and thinner growth plates.***A*, H&E stain, Safranin O/fast green stain of tibia sections. *B*, measurement of the thickness of metaphyseal zone and growth plate thickness of 2-month-old tibias. Results are expressed as mean ± SD, the *p*-values for statistical comparisons between groups are shown in the Figure, n = 5. *C*, immunohistochemistry analysis of tibia sections using the primary antibody of Col1α1 and Ihh (Indian hedgehog). The boxed regions in the previous row of pictures were magnified in the right pictures, respectively. Scale bar represents 200 μm, 50 μm, 20 μm, respectively. *D*, frozen sections showed that Cre-GFP was detected as *green* fluorescence in the tibia of *Sp7-Cre; Rosa26*^*-ZsGreen1*^ and *Sp7-Cre; Hdac4*^*fl/fl*^*; Rosa26*^*-ZsGreen1*^ mice at 2 months. *E*, tartrate-resistant acid phosphatase (TRAP) staining was performed on the tibial tissue of 2-month-old *Hdac4*^*fl/fl*^ and *Sp7-Cre; Hdac4*^*fl/fl*^ mice. *F*, quantification of the numbers of GFP-positive osteoblasts, TRAP-positive osteoclasts, and percentage of osteoclast surface in the proximal metaphysis. Results are expressed as mean ± SD, n = 4. The *p*-values for statistical comparisons between groups are shown in corresponding images.

To further analyze the growth retardation in *Sp7-Cre; Hdac4*^*fl/fl*^ mice, we initially stained the tibiae of 2-month-old mice with H&E and safranin O (Fig. 4*A*). Simultaneously, we examined the characteristics of chondrocyte differentiation and bone formation by assessing the expression of Col1α1 (an osteogenic-related gene to evaluate bone formation) and Indian hedgehog (Ihh) (a marker for chondrocyte pre-hypertrophy) protein. H&E staining revealed that *Sp7-Cre; Hdac4*^*fl/fl*^ mice at 2 months of age exhibited decreased ossification and endochondral bone formation, evident through a significant reduction in the thickness of the osteogenic zone and a decline in osteoblast density (approximately 50% reduction) in the chondro-osseous junction (Fig. 4*B*). Furthermore, we observed a reduction in Col1α1-stained cancellous bone in the metaphyseal region, indicating a significant decrease in ossification (Fig. 4*C*). Histological examination with safranin O staining revealed that the growth plates of the tibiae were considerably smaller, with almost no columnar arrangement of chondrocytes, replaced by hypertrophic chondrocytes (Fig. 4*A*). Subsequent immunohistochemistry (IHC) using anti-Ihh antibodies demonstrated a substantial presence of Ihh-positive chondrocytes in the mutant growth plate, indicating that the chondrocytes underwent hypertrophy (Fig. 4*C*). Furthermore, we utilized Rosa26^−ZsGreen1^ reporter mice to obtain *Sp7-Cre; Rosa26*^*−ZsGreen1*^ and *Sp7-Cre;Hdac4*^*fl/fl*^*; Rosa26*^*−ZsGreen1*^ mice for performing lineage-tracing studies. Frozen sections from 2-month-old mice revealed Sp7-Cre-positive (green) cells in the developing metaphyseal cancellous bone in both groups of mice; however, there were fewer sparsely scattered Sp7-positive cells in the chondro-osseous junction of the mutant tibia (Fig. 4, *D* and *F*). GFP^+^ cells accumulated in the growth plate cartilage of mutant mice, and these were Ihh-expressing chondrocytes. Tartrate-resistant acid phosphatase (TRAP) staining confirmed that bone resorption was affected in mutant mice with reduced osteoclast numbers and osteoclast surfaces (Fig. 4, *E* and *F*).

### Sp7-Cre–mediated Hdac4 deletion induced reduced gene expression of major bone matrix proteins and decreased angiogenesis in the metaphyseal region

**Figure 5:**
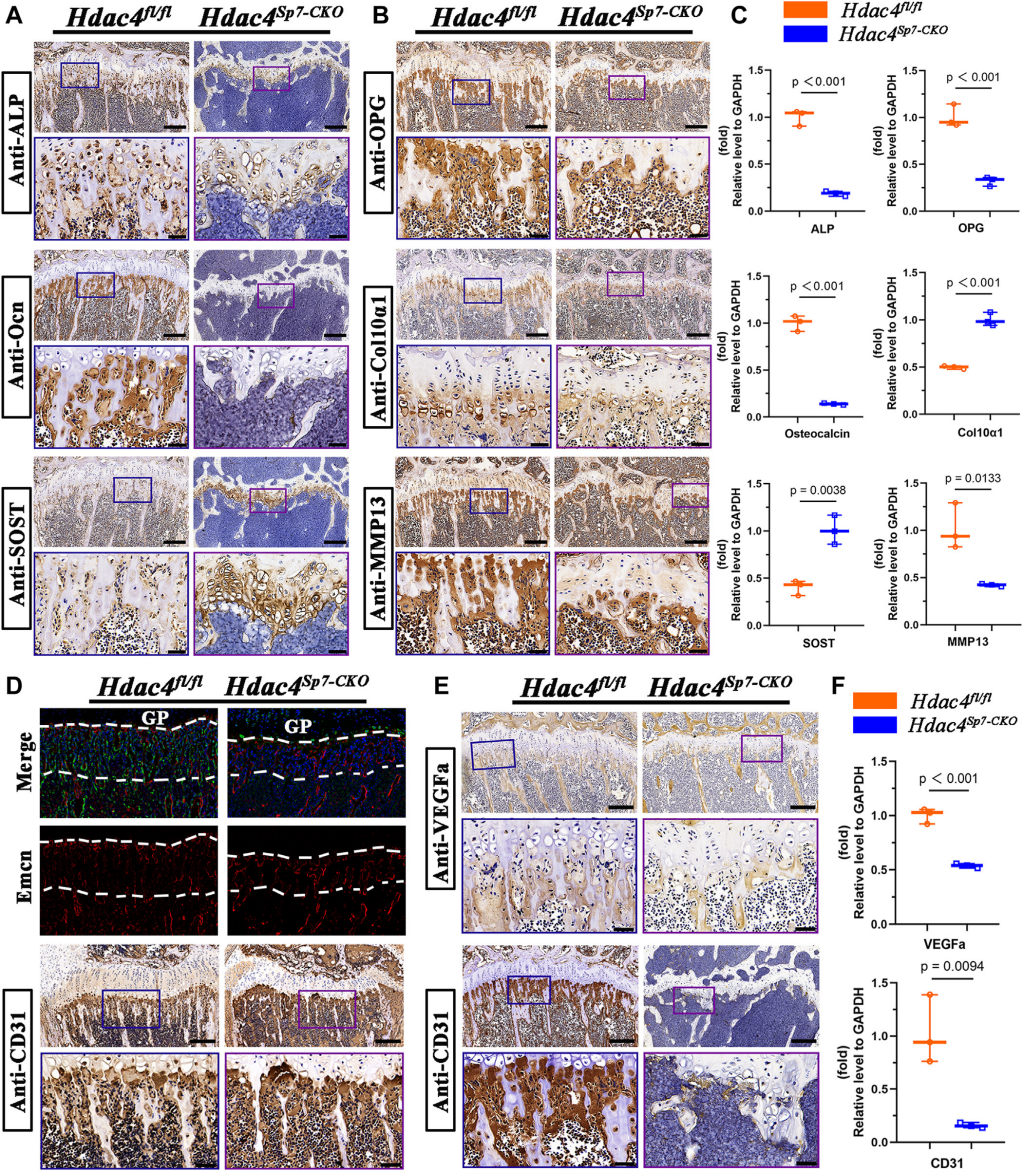
**Loss of Hdac4inSp7-expressing osteoprogenitors leads to reduced expression of major bone matrix proteins and decreased angiogenesis in the metaphyseal region.***A*–*C*, the protein expression profiles and gene expression levels of osteoblast and osteoclast markers ALP, osteocalcin, OPG, and SOST were examined using immunohistochemistry and RT-qPCR. Examination of protein expression profiles and gene expression levels of the markers of terminal chondrocyte differentiation (Col10α1, MMP13) using immunohistochemistry (IHC) and RT-qPCR. *D*, confocal images of immunofluorescence staining for Endomucin (Emcn) and Cre-GFP on frozen sections of the proximal tibia at P21; CD31 immunohistochemical staining at P21. *E*, CD31and VEGFa immunohistochemical staining of the proximal tibia in the experimental and control group at 2 months. *F*, the mRNA expression of angiogenesis-related genes (CD31,VEGFa) at 2 months was detected using RT-qPCR. n = 3, the *p*-values are shown in corresponding images. The values of *Hdac4*^fl/fl^ mice were defined as 1. Results are expressed as mean ± SD. Scale bars are 100 μm and 40 μm, respectively.

To elucidate the mechanism underlying the effects of HDAC4 on bone formation, we assessed the expression of bone matrix protein genes, including ALP, Col1α1, osteoprotegerin (OPG), sclerostin (SOST), and osteocalcin (Ocn), through IHC at 2 months of age. Additionally, we examined the expression of matrix metalloproteinase 13 (MMP13) and Col10α1 to evaluate the differentiation and degradation of cartilage cells (Fig. 5, *A* and *B*). Markers of osteoblast differentiation and activity, including ALP, Col1α1, and OPG, were weakly expressed in mutant mice, indicating impaired maturation and function of osteoblasts. Particularly, osteocalcin expression was nearly absent, indicating a severely impaired cellular mineralization capacity. In contrast, SOST, a negative regulator of bone formation, is abundantly expressed in the epiphysis and strongly expressed in mature hypertrophic chondrocytes and osteoblasts. MMP13 was abundantly detected in the metaphyseal region of 2-month-old *Ihh*^*fl/fl*^ mice, and the expression of these genes was weak in the primary spongiosa of the mutant mice. Sections from *Hdac4*^*fl/fl*^ mice at 2 months showed clear Col10α1 expression in hypertrophic chondrocytes, while sections from *Sp7-Cre; Hdac4f*^*l/fl*^ mice exhibited a broad pattern of Col10α1 expression, with expression in almost all growth plate chondrocytes. Real-time quantitative PCR (RT-qPCR) analysis of 2-month-old mice shows that the expression of ALP, Col1α1, OPG, MMP13, and osteocalcin was lower than that in *Hdac4*^*fl/fl*^ mice, whereas SOST (the gene encoding sclerostin) and Col10α1 were highly expressed (Fig. 5*C*).

Blood vessels beneath the growth plate are key components of the bone microenvironment that support the growth potential of perivascular cells, including bone progenitor cells and mature osteoblasts, thereby connecting osteogenesis with angiogenesis (20, 21, 22). Genetic lineage tracing revealed that GFP-positive cells are sparsely distributed in the metaphyseal region of 3-weeks-old *Sp7-Cre;Hdac4*^*fl/fl*^*; Rosa26*^*−ZsGreen1*^ mice, accompanied by a decreased density of Emcn^+^ vessels. The angiogenic marker (CD31) also exhibited a declining trend (Fig. 5*D*). At 2 months of age, we observed a weak expression of the angiogenesis-related marker Vegfa in the epiphyseal trabeculae immediately adjacent to the growth plate of *Sp7-Cre;Hdac4*^*fl/fl*^ mice. Furthermore, CD31 expression was barely detected in mutant tibias sections (Fig. 5*E*). RT-qPCR analysis of tibias revealed significantly reduced Vegfa and CD31 transcript levels in 2-month-old mutant mice compared to control littermates (Fig. 5*F*). The pronounced reduction in CD31 transcripts and the near absence of CD31-positive endothelium indicate impaired angiogenesis in the epiphysis, partially explaining the impaired trabecular bone formation beneath the growth plate.

### Sp7-Cre–mediated Hdac4 deletion leads to impaired differentiation and mineralization of osteoblasts *in**vitro***

**Figure 6:**
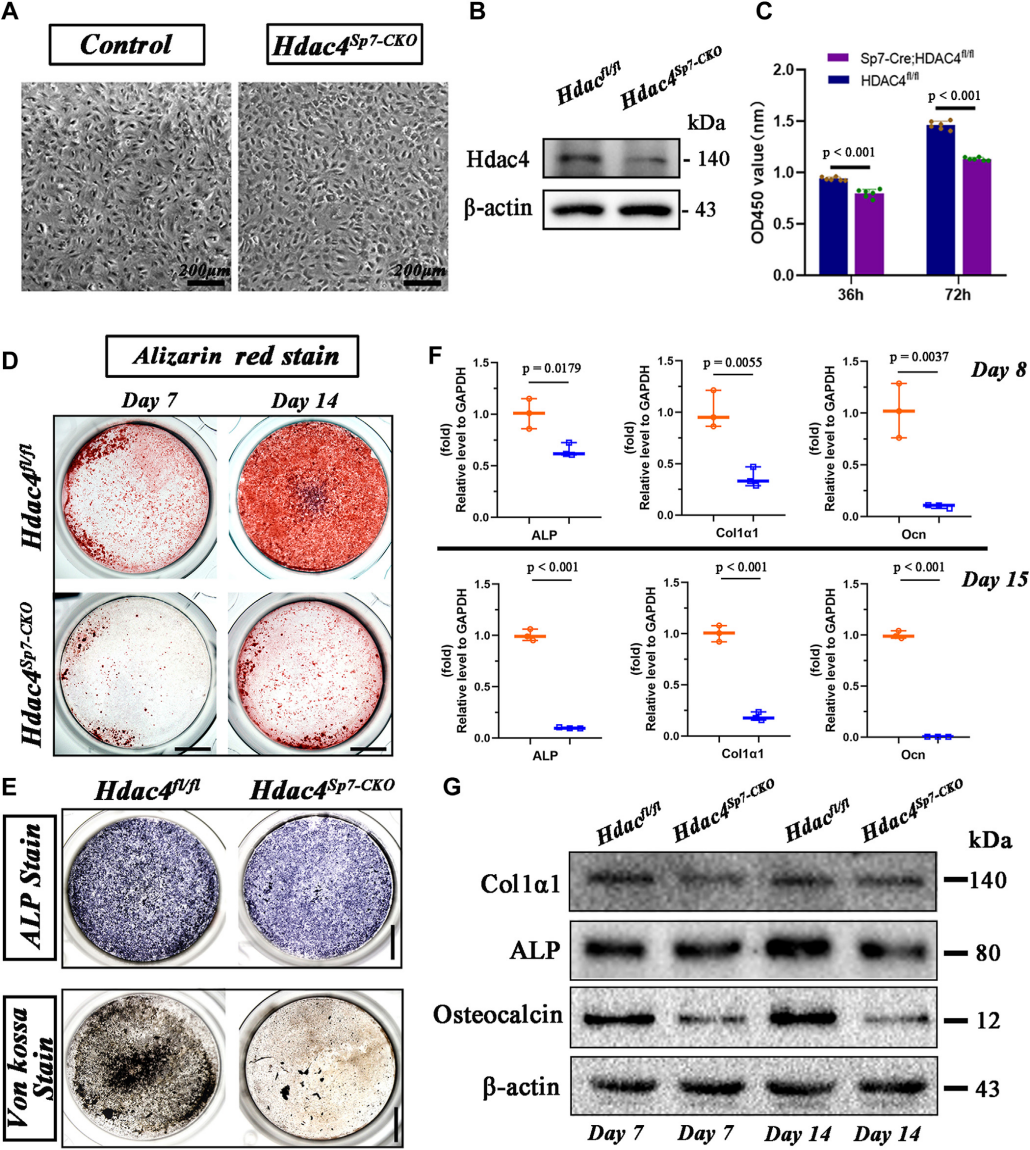
**Loss of Hdac4inSp7-expressing osteoprogenitors inhibited the differentiation and mineralization of osteoblasts *in vitro*.***A*, primary osteoblasts were isolated from the calvaria of postnatal days 6 to 7 *Hdac4*^*fl/fl*^ mice and *Sp7-Cre; Hdac4*^*fl/fl*^ mice and then cultured for proliferation and differentiation assays. *B*, relative to the *Hdac4*^*fl/fl*^ group, the protein level of Hdac4 decreased in the primary osteoblasts. *C*, primary osteoblast from calvarias of *Sp7-Cre; Hdac4*^*fl/fl*^ mice and control littermates were cultured for 36 h, 72 h in growth medium and then subjected to CCK8 assay. Results are expressed as mean ± SD, the *p*-values for are shown in corresponding images. *D*, alizarin red staining of primary osteoblast was performed after 6 and 12 days of culture in osteogenic medium, respectively. *E*, ALP staining and von Kossa staining of primary osteoblast were performed after 14 days of culture in osteogenic medium, respectively. Scale bars represent 5 mm. *F* and *G*, cells were harvested after 8 and 15 days of culture in osteogenic medium and subjected to RT-qPCR assay and Western blot assay to analyse the mRNA and protein levels of Col1α1, Alp, and Osteocalcin, respectively. Results are expressed as mean ± SD, n = 3. The *p*-values for are shown in corresponding images.

**Supplemental Fig. S2:**
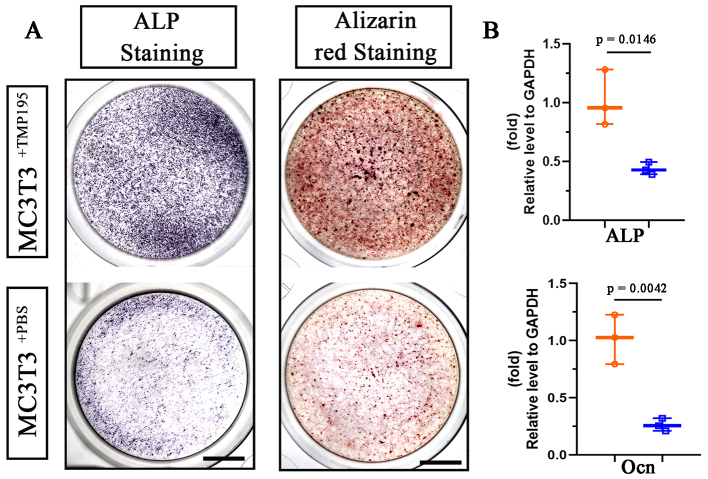


To determine the necessity of Hdac4 in osteoblast differentiation and mineralization, we initially treated mouse preosteoblast MC3T3-E1 cells with TMP195 (100 nM, dissolved in DMSO), a known inhibitor of HDAC4 function. After osteogenic induction for 5 days, we observed suppressed ALP activity and mineralization ability (ARS staining) in the presence of Hdac4 inhibition (Fig. S2*A*). Concurrently, the mRNA expression levels of ALP and osteocalcin were significantly reduced (Fig. S2*B*). After observing the role of Hdac4 in MC3T3-E1 cells, calvaria-derived primary osteoblasts from *Sp7-Cre;Hdac4*^*fl/fl*^ mice were isolated and cultured (Fig. 6*A*). Confirmation of Hdac4 deficiency in primary osteoblasts was achieved by assessing its protein expression (Fig. 6*B*). The CCK-8 assay indicated a relatively slow growth rate and inhibited the proliferative capacity of primary osteoblasts isolated from the mutant mice (Fig. 6*C*). Consistent with expectations, Hdac4-cKO osteoblasts exhibited decreased ALP activity (Fig. 6*E*). Moreover, impaired osteoblast mineralization capability was evident in primary osteoblasts from mutant mice, as shown by Alizarin Red S and Von Kossa staining (Fig. 6, *D* and *E*). RT-qPCR and western blotting (Fig. 6, *F* and *G*) revealed a significant decrease in the expression of molecular markers related to osteoblast differentiation. These *in vitro* experiments further supported the essential role of Hdac4 in modulating osteoblast function and mineralization.

### Sp7-Cre–mediated Hdac4 deletion alters the gene expression profile and affects multiple biological processes in RNA-seq analysis

**Figure 7:**
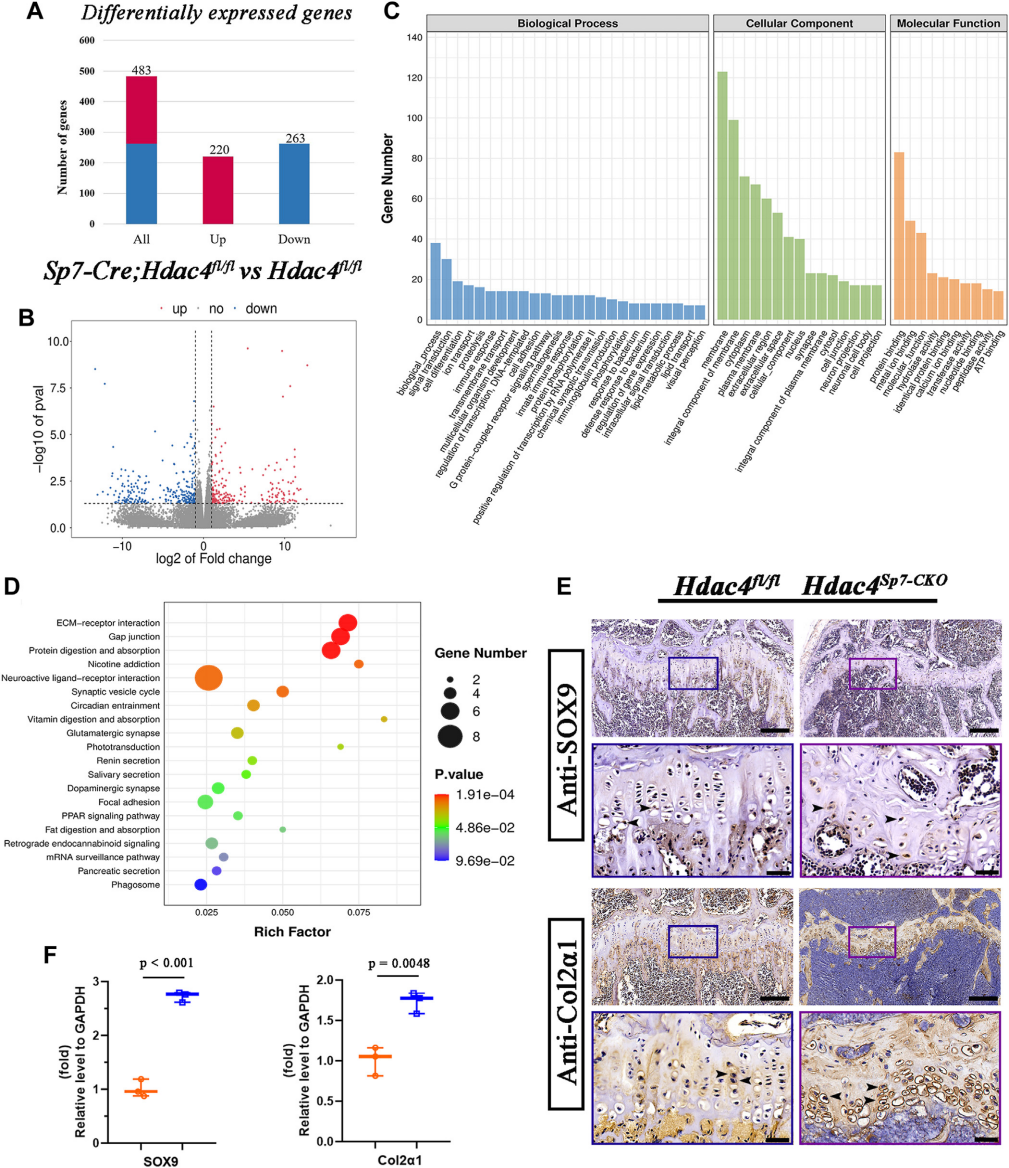
**Loss of Hdac4 in *Sp7*-expressing osteoprogenitors alters the gene expression profile and affects multiple biological processes.***A*, the bar chart shows the total differentially expressed genes (DEGs), upregulated DEGs, and downregulated DEGs. *B*, volcano plot of the differentially expressed genes (DEGs) for *Hdac4*^*fl/fl*^*versusSp7-Cre; Hdac4*^*fl/fl*^. *C*, gene Ontology (GO) functional enrichment analysis of all the DEGs for *Hdac4*^*fl/fl*^ and *Sp7-Cre; Hdac4*^*fl/fl*^ mice. *D*, Kyoto Encyclopedia of Genes and Genomes (KEGG) pathway enrichment analysis of all the differentially expressed genes (DEGs) for *Hdac4*^*fl/fl*^ and *Sp7-Cre; Hdac4*^*fl/fl*^ mice. *E*, immunohistochemistry analysis of tibia sections from *Hdac4*^*fl/fl*^ and *Sp7-Cre; Hdac4*^*fl/fl*^ at 2-month-old using the primary antibody of SOX9 and Col2α1. *F*, RNA isolated from control and mutant tibias at 2-month-old was subjected to RT-qPCR assay, and gene expression analysis of SOX9 and Col2α1 was conducted. All target genes' mRNA levels were normalized to Gapdh mRNA. Results are expressed as mean ± SD, n = 3. The *p*-values for are shown in corresponding images.

To delineate the potential molecular mechanism of Hdac4 in Sp7-expressing cells, RNA-seq was performed on tibias derived from either *Hdac4*^*fl/fl*^ or *Sp7-Cre; Hdac4*^*fl/fl*^ mice for an in-depth analysis of the genomic transcriptional network. The results of a volcano plot and differential gene expression analysis revealed that deletion of Hdac4 in Sp7-expressing cells led to substantial alterations in gene expression, with 220 genes significantly upregulated and 263 genes downregulated (log2FC > 2, *p* < 0.05) (Fig. 7, *A* and *B*). Gene Ontology enrichment analysis of all DEGs in the two groups of mice showed significant enrichment in multiple terms related to biological processes, cellular components, and molecular functions (Fig. 7, *C* and *D*). Figure 7*C* presents the top 20 Gene Ontology terms, including extracellular region, extracellular matrix, collagen type IX trimer, various biological processes related to signal transduction, lipoprotein metabolic processes, immunoglobulin production and immune response, guanylate cyclase activity, and cartilage development. Furthermore, the Kyoto Encyclopedia of Genes and Genomes analysis of all the DEGs highlighted enriched pathways, including “extracellular matrix-receptor (ECM-receptor) interaction,” “neuroactive ligand-receptor interaction,” “mRNA surveillance pathway,” “digestion and absorption,” “PPAR signaling pathway,” and “cAMP signaling pathway” (Figs. 7*D* and S3). Significantly, among the identified differentially expressed genes, upregulated Sox9 and Col2α1 were associated with cartilage development, serving as key regulators of early chondrocyte differentiation and cartilage-specific extracellular matrix genes, respectively. Recent studies have shown that Sox9 protects the spectral fate of chondrocytes by inhibiting their dedifferentiation, thereby maintaining the growth plate (23). Therefore, by IHC staining of tibial sections from two groups of mice, we further validated that the expression of Sox9 and Col2α1 was strongly observed in the tibial growth plate of 2-month-old *Sp7-Cre; Hdac4*^*fl/fl*^ mice (Fig. 7*E*). Subsequently, we collected tibias from 12-week-old *Hdac4*^*fl/fl*^ and *Sp7-Cre; Hdac4*^*fl/fl*^ mice and extracted total RNA for RT-qPCR analysis. Consistent with the IHC results, these data showed that the expression of Sox9 and Col2α1 at the mRNA level was significantly elevated in 12-week-old *Sp7-Cre; Hdac4*^*fl/fl*^ mice, respectively (Fig. 7*F*).

## Discussion

Osteoblasts are not only endocrine cells that contribute to whole-organism physiology but, more importantly, synthesize the extracellular bone matrix responsible for bone formation and regulate osteoclast differentiation involved in bone resorption (24, 25, 26). The class II HDAC molecule HDAC4 has been recognized for its importance in chondrocyte growth and bone formation since its initial characterization in Hdac4-deficient mice. However, subsequent expression profiles have also revealed that Hdac4 expression is higher in bone or osteoblasts than in other tissues, where it also performs important functions, such as in cartilage tissues (8, 14). Several studies have improved our understanding of the role of Hdac4 in osteoblast. For example, Hdac4 in osteoblasts can facilitate Rankl expression, thereby influencing osteoclast differentiation (14). Conditional deletion of Hdac4 in mature osteoblasts reduces cortical thickness and bone area (12), and mature osteoblasts and osteocyte-specific deletion in *Dmp1-Cre; Hdac4*^*fl/fl*^ mice showed no obvious skeletal phenotype (15). In this study, we established a novel transgenic mouse model where Hdac4 was predominantly deleted in Sp7-expressing osteoprogenitors. Our *in vivo* genetic investigations provide evidence that the deletion of Hdac4 in Sp7-expressing osteoprogenitors results in dwarfism and an osteoporotic bone phenotype. This phenotype is primarily characterized by impaired metaphyseal trabecular bone formation, thinner cortical bone, and a vestigial growth plate. Both *in vivo* and *in vitro* analyses revealed that the loss of Hdac4 does not affect prenatal bone development in *Sp7-Cre; Hdac4*^*fl/fl*^ mice. The second ossification center (SOC) develops normally, with the growth plate remaining intact during the early postnatal period. However, as these mice reach adulthood, severe impairments are observed in osteoblast differentiation, bone matrix protein secretion, angiogenesis, and sequential bone formation. Micro-CT analyses revealed a significant reduction in bone mineral density, trabecular bone volume, and cortical thickness owing to the loss of Hdac4. Importantly, this mouse model offers a unique opportunity for the continuous observation of Hdac4 function in bone development and homeostasis, spanning from the embryonic stage to postnatal life, leading to novel and distinctive findings.

Additionally, lineage-tracing studies have demonstrated that Sp7-expressing osteoprogenitors, along with blood vessels, migrate into the cartilage during embryonic development, forming trabecular bone. In the postnatal period, these osteoprogenitors are localized in the chondro-osseous junction, trabecular bone, and cortical bone (27, 28, 29, 30). Previous studies and ours have demonstrated the expression of Sp7-Cre in osteoblast progenitor cells at E14.5 (18, 19); however, we did not assess the role of Hdac4 in the early stages of skeletal development because no evidence of abnormal bone formation at birth was observed. During the postnatal formation of the SOC, our findings revealed a lack of cartilage canals invaginated from the perichondrium in *Sp7-Cre;Hdac4*^*fl/fl*^ mice at P7, indicating a delay in the invasion of osteoprogenitors and blood vessels into epiphyseal cartilage. Between 3 weeks to 2 months, the sustained loss of Hdac4 resulted in less trabecular bone, thinner cortical bone, and smaller growth plates in *Sp7-Cre;Hdac4*^*fl/fl*^ mice, contributing to dwarf and osteoporotic phenotypes. Endochondral ossification determines the longitudinal growth of long and trabecular bone formation but ultimately depends on osteoblast lineage cells for the deposition and mineralization of the bone matrix (31, 32, 33, 34). Hdac4 deficiency reduces the expression of several key factors that mediate the mineralization and activity of osteoblasts, suggesting that Hdac4 plays an important role in promoting the commitment of osteoprogenitors to osteoblasts, thereby maintaining trabecular and cortical bone formation. The development of POC in the postnatal tibia involves a novel mechanism of angiogenesis, specifically the extension of blunt vessel buds from vessel loops near the hypertrophic growth plate (35, 36). In this context, our study demonstrates that Hdac4 deficiency not only decreases the expression of genes related to angiogenesis but also diminishes the density of type H vessels in the chondro-osseous junction. This reduction in vessel density also contributes to the impairment of trabecular bone formation under the growth plate.

We also identified an interesting observation that *Sp7-Cre;Hdac4*^*fl/fl*^ mice exhibited a thinner growth plate with a slightly disordered chondrocyte arrangement during postnatal bone development, and nearly all of these chondrocytes expressed Ihh, a marker of prehypertrophic chondrocytes. In addition, RNA-seq analysis and further molecular biology experiments showed that Hdac4 deletion resulted in abundant expression of Sox9 and Col2α1 in mutant growth plates. Previous studies have demonstrated that Sp7-Cre is primarily detected in osteoblast precursors and their descendants, with sporadic lower-level expression in prehypertrophic chondrocytes (18, 19, 37). The prehypertrophic phase of chondrocytes is an important step in bone formation as the cells undergo a transformation into a mature phenotypic program; Sox9 was recently revealed to maintain the lineage fate of prehypertrophic cells and prevent the acquisition of an osteoblastic phenotype (38). Hence, we speculated that the observed growth plate phenotype may have resulted from perturbations in these cells and is directly or indirectly related to the Sox9 pathway, which needs to be elucidated by further studies. Additionally, our lineage-tracing studies indicated distinct Sp7-Cre–mediated GFP expression in the chondrocytes of growth plates but a significant reduction in GFP-positive osteoblasts in the osteogenic area. So, Hdac4 deficiency in Sp7-expressing osteoprogenitors might accelerate the transdifferentiation of growth plate chondrocytes into osteoblasts to support bone formation; however, these cells lacked the expression of osteogenesis-related genes and did not contribute to epiphyseal trabeculae formation. We speculated that this may be an alternative potential mechanism by which Hdac4 disruption leads to aberrant growth plate development. Follow-up work is needed to further explore the underlying, specific factors.

Notably, previous work reported by Emi Shimizu *et al.* has shown that Hdac4 in osteoblastic cells UMR 106-01 represses the expression of MMP13 (39). In another subsequent study, Nakatani T *et al.* showed that ablation of Hdac4 in 8-day-old mice resulted in increased MMP13 expression and protein levels *in vivo* (40). The findings described in this report indicate that deficiency of HDAC4 in Sp7-expressing osteoblasts resulted in the decreased expression of MMP13 at both the mRNA and protein levels by 2-month-old mice *in vivo*. Perhaps this difference is due to the deletion of HDAC4 in different tissues or cells and different periods of observation. Moreover, MEF2C, an important regulator of orchestrating transcriptional and cell-cell signaling events, is involved in chondrocyte hypertrophy during bone development (41). However, we did not delve into the effects of osteoblast-expressed Hdac4 on MEF2C and the underlying mechanisms in the present study. Although RNA-seq was performed to analyze the gene expression profile and potential pathways, a more in-depth elucidation of the molecular mechanisms through which Hdac4 affects bone development is necessary. For example, screening downstream relevant pathways regulated by HDAC4 and investigating their potential for targeted therapies in bone development or bone diseases and developing interventions targeting HDAC4 to promote bone formation or attenuate bone loss in osteoporosis. Due to the potential limitations of our mouse models, more targeted cell-specific deletion studies beyond osteoprogenitors and chondrocytes are need to explore the role of Hdac4 in other cell types within the bone microenvironment, such as mesenchymal cells, osteoclasts, or endothelial cells. These future studies can further expand our understanding of Hdac4's intricate role in bone biology and potentially uncover novel therapeutic avenues for bone-related disorders. In addition, the analyses focused on young adult mice, leaving the effects of Hdac4 deficiency during aging unexplored.

We also produced *Acan-Cre*^*ERT2*^*;Hdac4*^*fl/fl*^ mice, where Hdac4 was specifically removed from chondrocytes after birth. In contrast to *Sp7-Cre:Hdac4*^*fl/fl*^ mice, the phenotype of these mice resembled that of conventional Hdac4^−/−^ mice, displaying decreased proliferation of columnar chondrocytes, accelerated ossification, and premature closure of the growth plate. Furthermore, we also observed the normal formation of SOC and growth plates at P14; however, as development progressed, the growth plates gradually became thinner. At 3 weeks, we failed to observe the presence of growth plates but noted an increase in trabeculae in the metaphyseal region. These findings demonstrate that chondrocyte-expressed Hdac4 contributes decisively to the phenotype of Hdac4-null mice, promoting the elongation of endochondral skeletons by maintaining a normal growth plate. This observation differs from our findings in *Sp7-Cre:Hdac4*^*fl/fl*^ mice. Therefore, the phenotype of *Sp7-Cre:Hdac4*^*fl/fl*^ mice may result from perturbations within osteoblast lineage cells rather than in chondrocytes.

In conclusion, we successfully generated two mouse models (*Sp7-Cre; Hdac4*^*fl/fl*^ and *Acan-Cre*^*ERT2*^*; Hdac4*^*fl/fl*^) with deletion of Hdac4 in Sp7-expressing osteoprogenitors or chondrocytes and revealed that *Hdac4* deficiency increases susceptibility to dwarfism, growth plate closures, and osteoporosis progression. Differential phenotypes between *Sp7-Cre;Hdac4*^*fl/fl*^ and *Acan-Cre*^*ERT2*^*;Hdac4*^*fl/fl*^ mice add valuable insights, indicating that Hdac4 in Sp7-expressing osteoprogenitors mainly regulates osteoblast function and impairs trabecular and cortical bone formation, thereby influencing skeletal development and homeostasis during postnatal bones development under physiological conditions.

## Experimental procedures

### Generation and maintenance of two conditional Hdac4 KO mice

The animal experimental protocol adheres to the “Guidelines for the Care and Use of Laboratory Animals” published by the China Animal Research Council. The Ethics Committee of the Second Hospital of Shanxi Medical University (Approval No. DW2022075) approved all experiments. All mice were housed under a 12-h light/12-h dark cycle, with free access to standard laboratory food and water. The mice utilized in our study, including *Sp7-Cre* mice (Stock Number: 110131, Biocytogen Co, Ltd), *Acan-Cre*^*ERT2*^ mice (Stock Number: 019148, The Jackson Lab), and *Hdac4*^*fl/fl*^ transgenic mice (Biocytogen Co., Ltd), were used in our study, as previously described (8, 18, 42). Details on the specific breeding strategies for *Sp7-Cre; Hdac4*^*fl/fl*^ mice, *Acan-Cre*^*ERT2*^*;Hdac4*^*fl/fl*^ mice, and *Hdac4*^*fl/fl*^ mice are provided in the following sections. In addition, *B6.Cg-Gt (ROSA)26Sor*^*tm6(CAG−ZsGreen1) Hze*^ (Stock Number: 007906, The Jackson Lab) mice (43) were used to assess Cre-recombinase expression. Mouse-tail or toe samples were collected for genotype identification through conventional PCR. The activation of Cre recombinase expression in *Acan-Cre*^*ERT2*^; *Hdac4*^*fl/fl*^ mice was achieved by administering a tamoxifen solution (Sigma) at a working concentration of 20 mg/ml, dissolved in corn oil (44). Mice were injected with a single dose of 20 μl of tamoxifen solution or vehicle at P1.

### X-ray and micro-computed tomography analyses

Radiographic evaluations of whole-body or tibial changes in mutant and control mice were performed using a small-animal X-ray apparatus (Faxitron UltraFocus). The exposure time and kV were set to “fully automatic,” and whole-body or tibial X-rays were administered to mice after euthanasia *via* cervical dislocation.

For the analysis of cortical and trabecular bone, the right proximal tibia from each mouse was *ex vivo* scanned using a micro-computed tomography system (μCT80; ScancoMedical AG). The scanning procedure involved starting at the growth plate and extending distally, with a fixed isotropic voxel size of 10.1 μm (specific parameters: 70 kV, 113 mA, integration time 300 ms). Uniform parameters in the trabecular region of the tibia were assessed by initiating measurements 0.1 mm from the growth plate and extending 80 continuous slices distally. The evaluated parameters encompassed BV/TV (%), number of beams (Tb.N, mm^−1^), beam thickness (Tb.Th, mm), and beam spacing (Tb.Sp, mm). Morphological parameters of the cortical bone were determined using total cortical area (Tt. Ar, mm^2^), cortical bone area (Ct. Ar, mm^2^), cortical area fraction (Ct.Ar/Tt.Ar, %), and cortical thickness (Ct.Th, mm).

### Skeletal preparation, histological analysis, IHC, and TRAP staining of tibiae sections

Whole skeleton staining was performed following our previously modified staining method (19). For histological analysis and immunohistochemical staining, hindlimbs were harvested, fixed in 4% paraformaldehyde for 24 h at 4 °C, and subsequently decalcified using a 15% ethylenediaminetetraacetic acid ultrasonic decalcification instrument. Following paraffin immersion and embedding, 5-μm sections were cut for H&E staining or Safranin O staining to assess histomorphology. Von Kossa staining on undecalcified 8-μm tibial sections was performed using a commercial kit (G3282, Solarbio) according to the protocol of the manufacturer. In accordance with the instruction of the manufacturer, successive tibial sections underwent TRAP staining using a kit from Sigma-Aldrich. Immunohistochemical staining was used to detect the corresponding protein expression on decalcified 5-μm slides as previously described (19). Information on the primary antibodies used is provided in Table S2. Horseradish peroxidase–labeled goat anti-rabbit, or anti-mouse IgG, was used to conjugate the primary antibodies. The specific secondary antibody was detected using Fast 3,3′-diaminobenzidine chromogen (ZSGB-Bio). For frozen sections, the knee joints were harvested and fixed with 4% paraformaldehyde/0.1 M phosphate buffer for 16 h at 4 °C. After thorough rinsing in PBS solution, the samples were immersed in 30% sucrose at 4 °C until sinking. Subsequently, the specimens were embedded in an O.C.T. Compound (SAKURA 4583) and sectioned at 7-μm thickness using a Leica CM1950 cryostat.

### Cell culture, ALP activity, and Alizarin red staining assay

Mouse preosteoblast MC3T3-E1 cells (Procell CL-0378) for studying osteoblast differentiation were procured from *Procell Life Science& Technology Co, Ltd*. Mouse primary osteoblasts were isolated from the calvaria of *Hdac4*^*fl/fl*^ and *Sp7-Cre; Hdac4*^*fl/fl*^ mice on postnatal days 6 to 7, following established protocols (19). The collected cells, including preosteoblast MC3T3-E1 cells, were cultured in α-MEM supplemented with 10% fetal bovine serum, 100 U/ml penicillin, and 100 μg/ml streptomycin at 37 °C with 95% air and 5% CO_2_. When cells reached approximately 80% confluence, the differentiation culture medium, consisting of 10% fetal bovine serum, L-ascorbic acid, 10 mM glycerophosphate, 100 U/ml penicillin, and 100 μg/ml streptomycin, was introduced to induce cell differentiation. Alkaline phosphatase staining of the cells was performed using a BCIP/NBT ALP color development kit (Beyotime) according to the instructions of the manufacturer. For Alizarin red staining, a 1% ARS solution (pH 4.2; Sigma, A5533) was utilized. ALP-active cells or mineralized nodules were identified and captured with a zoom stereomicroscope (Leica M205).

### Western blotting and RT-qPCR

Bone tissue from mice was promptly frozen in liquid nitrogen upon harvesting and stored in a −80 °C refrigerator. Total protein extraction from bone tissues or cultured cells was performed using a Whole Cell Lysis Assay kit (KeyGEN BioTECH), according to the instructions of the manufacturer. Equal amounts of proteins were then separated *via* SDS-PAGE. Following that, they were transferred onto a polyvinylidene fluoride membrane. Subsequent to incubation with primary antibodies against Hdac4 (Abcolnal, A0239 or Cell Signaling Technology, 7628), Col1α1, ALP, osteocalcin, β-actin, and the corresponding HRP-labeled secondary antibody, protein bands were detected using a highly sensitive ECL chemiluminescence kit (Boster) and visualized with a Bio-Rad imaging system. To determine the expression levels of the genes of interest, reverse transcription RT-qPCR was performed. Total RNA was isolated from cultured cells or *in vivo* samples utilizing the TRIzol reagent (Invitrogen) according to the instructions of the manufacturer. The *in vivo* samples were obtained as described below. Following the reverse transcription of RNA to DNA using the Prime Script RT-PCR kit, gene expression analysis was performed using the QuantStudio 6 Flex Real-Time PCR System (Thermo Fisher Scientific). The mRNA levels of all related genes were normalized to the GAPDH expression level using the 2^−ΔΔCT^ method. Table S1 presents the primer sequences used for the RT-qPCR.

### RNA extraction and RNA-seq analysis

RNA was isolated from murine bone tissue using TRIzol reagent (Invitrogen) according to the protocol of the manufacturer. Specifically, hindlimb tibias from both groups of mice at 4 weeks of age were harvested, rid of all incidental muscle tissue, and centrifuged (12,000 rpm/min × 5 min) at 4 °C using an ultrahigh-speed centrifuge to remove bone marrow. The tibias were then incised through the metaphysis and distal TFJ to isolate the area of interest for the bone tissue. The entire isolation process was performed on ice, and the samples were rapidly frozen in liquid nitrogen for 30 min and stored in the refrigerator at −80 °C. Subsequently, total RNA was isolated and purified using the TRIzol reagent (Invitrogen) following the instruction of the manufacturer. RNA concentration was determined using a Nanodrop (ND-2000; Thermo Fisher Scientific). RNA integrity, with a RIN number >7.0, was assessed using a Bioanalyzer 2100 (Agilent) and confirmed by electrophoresis on a denaturing agarose gel. RNA-seq procedures and initial analyses were performed by LC Bio Technology, involving 2 × 150 bp pairwise end-to-end sequencing on an Illumina Novaseq 6000 (PE150). The mRNA expression levels were calculated using FPKM (total_exon_fragments per mapped_reads [millions] values) and estimated using the StringTie software (https://ccb.jhu.edu/software/hisat2). Differentially expressed mRNAs were identified based on a fold-change of >2 or <0.5, along with a parametric F-test comparing the nested linear models (*p* < 0.05).

### Statistical analysis

Data obtained from three or more independent experiments are presented as the mean ± SD. Statistical analyses were performed using SPSS software or GraphPad Prism. Between-group comparisons utilized Student’s t-tests, while multiple-group comparisons used one-way ANOVA. Statistical significance was set at *p* < 0.05.

## Data availability

All data are contained within the manuscript and/or in the Supporting information that are provided. RNA-Seq data were deposited in the GEO public data repository, with the following link “https://www.ncbi.nlm.nih.gov/sra/PRJNA1100183”.

## Supporting information

This article contains supporting information.

## Conflict of interest

All authors declare that they have no conflicts of interest with the contents of this article.

## Figures and Tables

**Supplemental Fig. S3:**