# Supplementary material for: Loss of Hdac4 in osteoprogenitors impairs postnatal trabecular and cortical bone formation, resulting in a dwarfism and osteopenia phenotype in mice
Source: J Biol Chem. 2024 Oct 29;300(12):107941. doi: 10.1016/j.jbc.2024.107941 (PMC11664417; doi:10.1016/j.jbc.2024.107941)
Supplement: Supplemental Table S1 [file mmc1.docx]

**Table 1 A. Primer sequences for Cre transgene and floxed Hdac4 genes**

| **Gene name** | **Primer sequence** | **Product size** |
| --- | --- | --- |
|  | F, 5′‐TACCAGAAGCGACCACTTGAGC‐3 | WT: 263bp |
| *Sp7-Cre* | WT-R, 5′‐CGCCAAGAGAGCCTGGCAAG‐3′ | Cre: 445bp |
|  | Cre-R, 5′‐GCACACAGACAGGAGCATCTTC‐3′ |  |
| *Hdac4*-flox | F, 5′-ATCTGCCCACCAGAGTATGTG-3′,  R, 5′-CTTGTTGAGAACAAACTCCTGCAGCT-3′ | WT: 480bp  Floxed Hdac4：620bp |
| *Acan-Cre^ERT2^* | F, 5′-GTTATATTCCGGAGCCCA CA -3′ | WT: 299bp |
|  | WT-R, 5′-AAAAGCGACAAGAAGACACCA-3′ | Cre: 200bp |
|  | Cre-R, 5′-CTCCAGACTGCCTTGGGAAAA-3′ |  |

**Table 1 B. Information of the primers used for RT-qPCR analysis**

| **Gene name** | **Forward primers (**5′-3′**)** | **Reverse primers (**5′-3′**)** |
| --- | --- | --- |
| *Hdac4* | GGCGAGCACAGAGGTGAAGATG | GCTGTGCTGTGTCTTCCCATAC |
| *MMP13* | GGACCTTCTGGTCTTCTGGC | GGATGCTTAGGGTTGGGGTC |
| *Col1α1* | TGAACGTGGTGTACAAGGTC | CCATCTTTACCAGGAGAACCAT |
| *Osteocalcin* | GGACCATCTTTCTGCTCACTCTGC | TCCTGCTTGGACATGAAGGCTTTG |
| *ALP* | TGACCTTCTCTCCTCCATCC | CTTCCTGGGAGTCTCATCCT |
| *Sost* | TCCTGAGAACAACCAGACCA | GCAGCTGTACTCGGACACATC |
| *Col10α1* | GCCAGGAAAGCTGCCCCACG | GAGGTCCGGTTGGGCCTGGT |
| *Col2α1* | CGGTGGTACGGTGTCAGG | TTATACCTCTGCCCATTCTGC |
| *SOX9* | CGTGGACATCGGTGAACTGA | GGTGGC AAGTATTGGTCAAACTC |
| *Vegfa* | ACCAGCGCAGCTATTGCCGT | CACCGCCTTCTTGGCTTGTCACA |
| *CD31/Pecam1* | CGTATCCAAGCCCAAGGTGA | GCACAAAGTTCTCGTTGGAGG |
| *GAPDH* | ACTGAGGACCAGGTTGTC | TGCTGTAGCCGTATTCATTG |
| *OPG* | ATGAACAAGTGGCTGTGCTGC | GGCAAGGGACACACAATGTC |
