# Supplementary material for: Loss of Hdac4 in osteoprogenitors impairs postnatal trabecular and cortical bone formation, resulting in a dwarfism and osteopenia phenotype in mice
Source: J Biol Chem. 2024 Oct 29;300(12):107941. doi: 10.1016/j.jbc.2024.107941 (PMC11664417; doi:10.1016/j.jbc.2024.107941)
Supplement: Supplemental Table S2 [file mmc2.docx]

**Table 2**

**Table 2 Primary antibodies, stock number and dilutions**

| **Antibody name** | **Manufacturer/Stock Number** | **Dilution** |
| --- | --- | --- |
| Anti-Hdac4 | Sangon Biotech/D160482 | 1:50 (IHC) |
| Anti-Hdac4 | Cell Signaling Technology(7628) | 1:1000 (Western blotting) |
| Anti-Hdac4 | Abclonal/A0239 | 1:1000 (Western blotting) |
| Anti-Ihh | Abcam/ab39634 | 1:100 |
| Anti-Col1α1 | Abclonal/A16891 | 1:100 |
| Anti-Osteocalcin | Santa Cruz/sc-365797 | 1:100 |
| Anti-Col2α1 | Abcam/ab34712 | 1:200 |
| Anti-Col10α1 | Abcam/ab58632 | 1:100 |
| Anti-Opg | Bioss/bs-0431R | 1:100 |
| Anti-Vegfa | Abclonal/A12303 | 1:100 |
| Anti-CD31 | Santa Cruz/sc-376764 | 1:200 |
| Anti-SOST | Abclonal/A8213 | 1:100 |
| Anti-MMP13 | Abcam/ab39012 | 1:200 |
| Anti-ALP | Abclonal/A0514 | 1:100 |
| Anti-SOX9 | Boster/BM4268 | 1:50 |
| Anti-Endomucin | Abclonal/A22662 | 1:100 |
