# Supplementary material for: Loss of Hdac4 in osteoprogenitors impairs postnatal trabecular and cortical bone formation, resulting in a dwarfism and osteopenia phenotype in mice
Source: J Biol Chem. 2024 Oct 29;300(12):107941. doi: 10.1016/j.jbc.2024.107941 (PMC11664417; doi:10.1016/j.jbc.2024.107941)
Supplement: Supplemental Figs. S1–S3 Legend [file mmc3.docx]

**Fig. S1** Loss of Hdac4 in *Sp7*-expressing osteoprogenitors generally has no effect on prenatal bone development. **A.** Several types of transgenic mice genotyping by conventional PCR using the specific primers listed in supplemental table S1. Upper panel: wild-type Hdac4^fl/fl^ (480bp); heterozygous Hdac4^fl/fl^ (480bp and 620bp); homozygous Hdac4^fl/fl^ (620bp); Sp7-Cre (445bp). Lower panel: wild-type Hdac4^fl/fl^ (480bp); heterozygous Hdac4^fl/fl^ (480bp and 620bp); homozygous Hdac4^fl/fl^ (620bp); Acan-Cre^ERT2^ (200bp). **B.** The mRNA level of Hdac4 in the lungs and kidneys of Hdac4^fl/fl^ and Sp7-Cre; Hdac4^fl/fl^ mice was measured by RT-qPCR using primers as shown in supplemental table S1. Western blotting also confirmed that Hdac4 in the kidneys and lungs of mutant and control mice were comparable in protein levels. **C.** Histologic analysis of tibial phenotype of P1 mice by H&E staining and Von Kossa staining showed a generally normal phenotype. Bar = 100μm. **D.** Measurement of body weight in mice of different ages. n= 4. Results are expressed as mean ± SD, the p-values for are shown in corresponding images.

**Fig. S2** Inhibition of HDAC4 in vitro affects ALP activity and mineralization in mouse pre-osteoblast MC3T3-E1 cells. **A.** ALP staining and von Kossa staining of MC3T3-E1 cells were performed after 5 days of culture in osteogenic medium, respectively. Scale bars = 5 mm **B.** RT-qPCR assay to analysis the mRNA levels of ALP and osteocalcin. Results are expressed as mean ± SD, n = 3. The p-values for are shown in in corresponding images.

**Fig. S3** KEGG pathway enrichment analysis of DEGs between the Hdac4^fl/fl^ mice and Sp7-Cre; Hdac4^fl/fl^ mice at 4 weeks of age.
